# Mapping leadership, communication and collaboration in short-term distributed teams across various contexts: a scoping review

**DOI:** 10.1136/bmjopen-2023-081878

**Published:** 2024-10-23

**Authors:** Hanna Morian, Johan Creutzfeldt, Magnus Hultin, Maria Härgestam

**Affiliations:** 1 Department of Nursing, Umeå University, Umeå, Sweden; 2 Clinical Science, Intervention and Technology, Karolinska Institutet, Stockholm, Sweden; 3 Department of Surgical and Perioperative Sciences, Umeå University, Umeå, Sweden

**Keywords:** Health & safety, Human resource management, Quality in health care

## Abstract

**ABSTRACT:**

**Introduction:**

Increased globalisation and technological advancements have led to the emergence of distributed teams in various sectors, including healthcare. However, our understanding of how leadership, communication and collaboration influence distributed healthcare teams remains limited.

**Objectives:**

This study aimed to map knowledge on leadership, communication and collaboration in short-term distributed teams across various fields to gain insights that could benefit healthcare.

**Design:**

Scoping review.

**Data source:**

A database search of PubMed, CINAHL, Scopus and PsycINFO was conducted in May 2021 and updated in February 2023 and May 2024.

**Eligibility criteria:**

Articles were eligible if they involved leadership, communication or collaboration in distributed short-term teams supported by synchronised audio-visual communication technology. Two researchers independently screened titles, abstracts and full texts for inclusion.

**Data extraction and synthesis:**

Extracted data on leadership, communication and collaboration were synthesised narratively and reported in terms of patterns, advances, gaps, evidence for practice and research recommendations.

**Results:**

Among 6591 articles, 55 met the eligibility criteria, spanning military, engineering, business, industrial and healthcare contexts. The research focus has shifted over time from adverse effects to solutions for overcoming challenges in distributed teams. Inclusive leadership is vital for engaging all team members. ‘Team opacity’, the absence of non-verbal cues and reduced awareness of team members’ actions, can occur in distributed teams relying on technology. Clear communication is crucial for avoiding misunderstandings and fostering collaboration and adaptability. Developing shared mental models and trust is more challenging, leading to uncertainty and reduced information sharing. There is a lack of studies examining how to apply this knowledge to health professionals’ education.

**Conclusion:**

Our findings highlight the importance of implementing strategies in healthcare to enhance inclusive leadership and improve communication in distributed healthcare settings. More empirical research is needed to understand the intricacy of distributed healthcare settings and identify effective ways to train distributed healthcare teams.

**Strengths and limitations of this study**

- The study followed the methodological framework of Arksey and O’Malley.
- Guided by a research librarian, a thorough database search was performed and updated twice before publication.
- Searching a wide variety of contexts allowed for identifying more knowledge on this topic; however, contradictory results may arise when researching different contexts and teams.
- Since this scoping review was exploratory in nature, no formal risk of bias assessment was performed.

## Introduction

 Globalisation and technological advancements have allowed organisations to manage more complex remote work environments.^1^,^4^ In distributed teams, members are geographically dispersed and use various technologies to collaborate.^5^ Such teams are becoming increasingly common across various sectors, for instance, in industry,^6^ business^7^ and the military.^8^ Distributed settings are also employed to organise the workforce when facing emergencies such as the COVID-19 pandemic, during which healthcare systems were forced to evolve and adjust.^9 10^ In some contexts, distributed teams became the only viable option for providing healthcare during the pandemic.^11^ This marks an important shift from traditional healthcare team models that rely on co-located teams.^12 13^

Unlike other sectors, healthcare faces unique challenges when implementing distributed teams. These challenges include the need for real-time decision-making under time pressure, the critical responsibility of patient care and the formation of ad hoc teams.^14^ Healthcare is a dynamic, high-risk environment where team members must quickly adapt, manage complex tasks and make critical decisions based on rapidly available information. Non-technical skills like leadership and communication are essential for maintaining safety and efficiency.^15^ Effective teamwork is crucial for optimising patient outcomes and ensuring safety, as these teams must perform at the highest level.^16 17^

Extensive research has explored the characteristics of well-functioning teamwork. The literature offers several models for traditional team settings where team members are physically present (ie, co-located).^18^,^22^ In such cases, effective teamwork in traditional co-located teams depends on communication, shared commitment, organisational support and resources.^18 23^ Team leadership is widely acknowledged as crucial, with multiple meta-analyses showing that it significantly impacts team success.^24 25^ Understanding the dynamics of leadership, communication and collaboration is foundational for ensuring effective teamwork in co-located teams, and there is no evidence that distributed teams differ in this respect.^19 24 26^ Focusing on these aspects aligns with their established importance in traditional co-located team settings.^19 24^

Research on human performance and teamwork in distributed teams has been ongoing for years, and various stakeholders and researchers worldwide have studied the topic.^27^ However, despite its increased occurrence in healthcare, there is a lack of research on teamwork in distributed healthcare teams.^28^ According to media richness theory, which posits that communication effectiveness is enhanced when richer media convey social cues and provide immediate feedback, the complexities of teamwork in distributed healthcare teams may not be fully understood.^29^ We therefore conducted a scoping review (ScR) in order to map knowledge on leadership, communication and collaboration in short-term distributed teams across various fields. This broader perspective could support adaptation in distributed healthcare teams and encourage further research.

## Methods

### Design

In this ScR, we used the methodological framework described by Arksey and O’Malley,^30^ which involves five sequential stages: (1) identifying the research question; (2) identifying relevant articles; (3) selecting articles; (4) charting the data; and (5) collating, summarising and reporting the results. The Preferred Reporting Items for Systematic Reviews and Meta-Analyses extension for Scoping Reviews (PRISMA-ScR) checklist^31^ was followed to ensure transparent and comprehensive reporting (see online supplemental material 1).

### Identifying the research question

This study aimed to map knowledge on leadership, communication and collaboration in short-term distributed teams across various fields. Once the lack of research within healthcare became clear, a contextual revision was required to encompass a wider setting with distributed teams in various contexts. Widening the area of interest for the investigation had methodological implications. An ScR can be helpful on a topic that is extensive and exhibits a complex or heterogeneous nature^30^; thus, this review type was chosen for the present study. The following research questions on leadership, communication and collaboration in distributed teams were probed to guide this ScR:

1. What patterns related to leadership, communication and collaboration in distributed teams have been reported?
2. What progress has been made in research within the publication period of the included studies?
3. What knowledge gaps exist in the research field of leadership, communication and collaboration in distributed teams?
4. Which results from research on leadership, communication and collaboration in other distributed contexts can be applied to the field of healthcare?

### Identifying relevant articles

A comprehensive initial search was undertaken in May 2021, followed by updated searches in February 2023 and May 2024 to ensure the inclusion of the latest publications. With assistance from a research librarian, we searched PubMed, CINAHL, Scopus and PsycINFO. Our database searches were not restricted by specific publication years or types; instead, we conducted unrestricted searches to ensure comprehensive coverage of the available literature. Search strings were developed for each database, including the keywords Distributed team, Virtual team, Ad hoc team, Teamwork, Leadership, Communication and Collaboration. The full search queries are given in online supplemental material 2. Manual searches for relevant publications were also made to ensure comprehensiveness.

### Selecting articles

Search results were imported into EndNote. After removing duplicates, articles were screened using Rayyan (title and abstract) and Covidence (full text) software. The following inclusion criteria were applied: English or Swedish language; focus on key teamwork concepts of leadership, communication or collaboration; at least one team member was located at a distance; teamwork was supported by synchronised audio-visual communication technology; and teams collaborated only in the short term (<24 hours). The short-term criterion was chosen based on our observations during the data search preparation. We noted that many distributed teams in various contexts operated over longer durations and hence could be considered to be established teams. This contrasts with healthcare teams, which are often formed for short-term tasks. Short-term teams, consisting of personnel who have not collaborated before and who plan to disband after finishing the task, work differently from established teams,^32^ and it is essential to draw parallels in teamwork aspects commonly found in short-term teams.

Each article was screened independently by at least two authors. The titles and abstracts were first reviewed, followed by a full-text screening against the eligibility criteria. Discrepancies were resolved with discussion or the involvement of a third author. If full texts were not retrievable, corresponding authors were contacted. In cases where the technology or the working period could not be discerned from the title or abstract, the articles were included for full-text screening. Articles lacking abstracts were also included for full-text screening.

### Charting the data

An extraction template in Covidence was designed according to the aim of this review and formed the basis for the tables and the reporting of the results. Two authors (HM and MHä) independently extracted author, title, year, country, design, setting, aim and findings on relevant teamwork concepts from the articles, and HM rechecked this for errors and completeness.

### Collating, summarising and reporting the results

The extracted data were narratively synthesised after being read multiple times by all four authors. Reporting on the synthesised data was guided by the five domains of the Patterns, Advances, Gaps, Evidence for practice and Research recommendations (PAGER) framework.^33^ Online supplemental material 3 provides a summary of our findings in terms of the PAGER domains: patterns of leadership, communication and collaboration (Patterns); advances made in the field (Advances); reflections on the paucity of research (Gaps); practical implications for distributed health professionals and organisations using distributed teams (Evidence for practice); and suggested areas for future research (Research recommendations).

### Patient and public involvement

Neither patients nor the public were involved in the design, conduct, reporting or dissemination plans of this research.

## Results

In this section, we first describe the characteristics of the included articles and then address the findings related to our research questions in more detail. Elements of the PAGER framework^33^ are integrated into the text under each research question to provide a comprehensive understanding of the findings.

### Characteristics of the included articles

**Figure 1:**
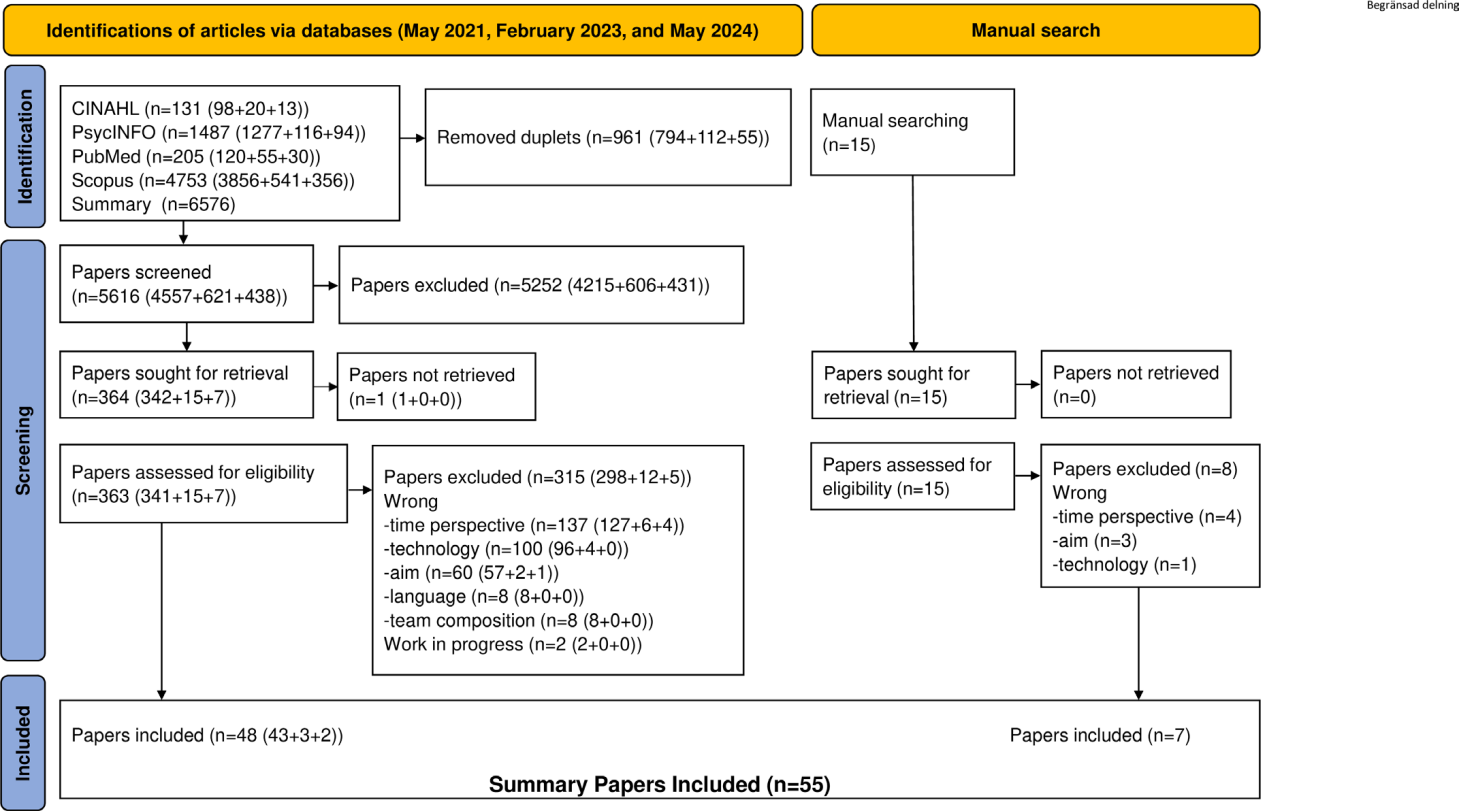
Preferred Reporting Items for Systematic Reviews and Meta-Analyses flow chart. Identifying, screening and including articles in the initial, updated and manual searches.

After screening, 55 out of 6591 publications were found to be eligible (figure 1). They were published between 2001 and 2023 and comprised journal articles, book chapters, conference papers, systematic reviews and theses (table 1); here, we use the word *articles* as a generic term to cover all of these. The articles had heterogeneous settings, including military, engineering, business and industrial; nine covered healthcare.

**Table 1:** Distribution of article types over the period 2001–2024*

Type of article	2001–2024n=55	2001–2008n=18	2009–2016n=22	2017–2024*n=15
Journal article	26	7	10	9
Book chapter	14	7	5	2
Conference paper	9	4	3	2
Systematic review	4	0	2	2
Thesis	2	0	2	0

* No articles from 2024 were included.

**Figure 2:**
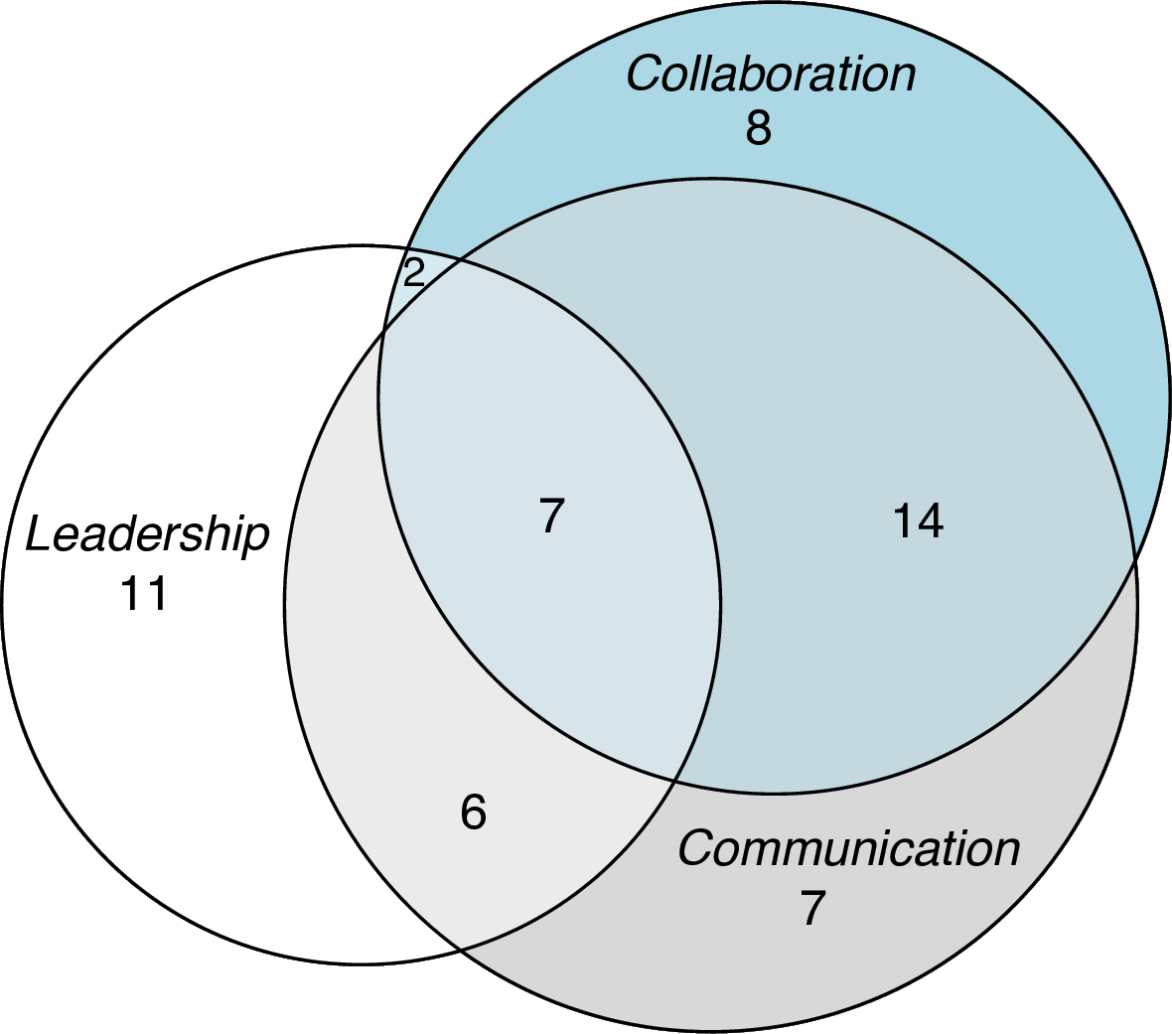
Key teamwork concepts examined in the included articles: leadership aspects (n=26), communication aspects (n=34) and collaboration aspects (n=31). These concepts are somewhat interdependent and overlapping.

Most contributions were from the USA, while the remainder were from Europe, Asia and Canada. The majority of articles covered only one or two teamwork concepts (ie, leadership, communication or collaboration); those that examined all three concepts were less common (figure 2). Research conducted with students in a laboratory was more common than empirical research, and the latter type was generally published in the past 5 years. Some of the articles lacked a clear methodological explanation. Details and characteristics of the articles are given in online supplemental material 4.

#### Terminology usage and definition

The articles used terminologies such as distributed teams (n=2), geographically distributed teams (n=3), dispersed teams (n=5), virtual teams (n=39), global virtual teams (n=2) and e-teams (n=1). Although ‘virtual teams’ was the most common terminology, we use the term ‘distributed team’ since it strongly connects to the geographical distribution of team members. The earlier articles had no working definitions of virtual or distributed teams. Still, over time, a general understanding was established in the literature that the members of these teams are located in more than one physical location and are connected through collaboration technologies.^34^ In the articles, team structure ranged from entirely distributed teams, where all team members were in different locations, to partially distributed teams with isolated and co-located subgroups of different sizes.^35^ Distributed team structure and context were described as dynamic, in the sense that team members assumed multiple roles and sometimes simultaneously managed various tasks.^35^

#### Characteristics in the medical context

A subset of the included articles (n=9) focused specifically on healthcare settings. These articles were published between 2008 and 2021 and exhibited some recurring themes and characteristics. Several articles focused on simulations or experiments involving healthcare professionals. For instance, Bolle *et al*^36^ and Butler *et al*^37^ conducted simulations with professionals from Norway and the USA, respectively, while Fang *et al*^38^ and Lazzara *et al*^39^ conducted randomised studies with US healthcare professionals. Xiao *et al*^40^ performed an experimental study to analyse remote leadership of real-life trauma resuscitation teams in a medical setting in the USA. Editorials by Kennel *et al*^28^ and White *et al*^41^ offered insights and reflections on the impact of distributed healthcare teams during the COVID-19 pandemic. Alhawary^34^ explored virtual teamwork within the Jordanian Royal Medical Services, whereas Keijser *et al*^42^ reviewed current healthcare research.

### Patterns related to leadership, communication and collaboration in distributed teams

Several articles (n=26) focused on the relationship between leadership and team performance in distributed teams. Half of the included articles (n=34) underscored the vital role of clear communication in enhancing performance. Studies on collaboration (n=31) explored a range of factors influencing team performance. While these articles often focused on specific themes, they frequently discussed overlapping leadership, communication and collaboration aspects.

#### Leadership in distributed teams

A significant portion of articles on leadership focused on leadership styles such as transformational, transactional, emergent, shared, task-focused and relationship-focused leadership, emphasising how context, task and team size influenced the appropriate leadership approach.^4041 43^,^53^ Leaders who combined hierarchical and encouraging behaviours were considered more effective as leaders.^28 40 45 47 49^ On the other hand, hierarchical leadership in distributed teams was found to be challenging due to the lack of social cues, underscoring the importance of adapting leadership styles in the distributed context.^54^ Some of the articles discussed how communication affected the suitability of leaders,^45 49 55^ with individuals who communicated more being suggested to be more suitable leaders.^45 47 49 54 55^

#### Communication in distributed teams

Communication delays and misunderstandings were frequently encountered in distributed settings.^53 56^ Comparisons of various communication technologies did not necessarily exhibit uniform outcomes.^57^ For instance, one study found that videoconference conversations were perceived as more polite and less disruptive, even though they may slow communication.^58^ In contrast, another study reported that videoconferencing might lead to less frequent communication compared with co-located teams.^52^ In distributed teams, if some members dominated communication, this could reduce the benefits of having larger teams, as other members might be marginalised and unable to contribute.^59 60^

#### Collaboration in distributed teams

When relying heavily on technology, distributed teams could experience ‘team opacity’, that is, a lack of non-verbal cues and a decreased awareness of team members’ actions. This increased workload and might obstruct collaboration, coordination and adaptability, all behaviours critical to performance.^26^ Communication difficulties within teams using technology tended to fade as the team spent more time working together. However, this adaptation benefited established teams rather than short-term ones.^58^

Familiarity among distributed team members was found to improve their effectiveness.^59 61^ Established teams working together for extended periods had an advantage due to their familiarity, which could be more difficult to establish for short-term teams.^59 61^ Similarly, building trust and a shared mental model was more challenging in distributed settings than in co-located teams.^4349 61^,^65^ Social cues were more readily available in co-located teams, affecting how much team members participated and paid attention in distributed teams.^2652 60 66^,^68^ While ‘getting to know each other’ meetings have been recommended as a way to overcome these challenges,^59 69^ this may not be feasible for short-term teams.

#### Distributed team aspects in medical contexts

Remote leaders in medical trauma teams were found to promote a hierarchical structure by communicating more with senior members, who then relayed information to junior members.^40^ In more urgent tasks, communication from distant leaders to the rest of the team increased, and the leader gave more direct instructions than otherwise.^40 50^

Overall, the distribution of team members across different locations did not positively or negatively affect collaboration during mass casualty simulation and trauma intensive care studies.^39 70^ However, in a study where real-time audio-visual communication was established between a neonatologist and a bedside provider, the efficiency and promptness of neonatal resuscitation scenarios improved. This type of video assistance ensured that the providers adhered more closely to the Neonatal Resuscitation Programme algorithm and established effective ventilation more rapidly compared with those who worked independently.^38^ Additionally, in the medical context, videoconferencing enhanced clinical work processes by promoting interaction between team members, leading to better multitasking and a shared mental model. Specialists were also more actively engaged when visually observing the patient, resulting in better treatment outcomes.^36^

### Progress in research over 20 years

Leadership in distributed teams has evolved significantly, according to research reported in these articles spanning the past 20 years. Earlier publications emphasised the lack of non-verbal cues, low cohesion, difficulty building relationships and low skills in enabling technologies.^44 71 72^ During the past decade, research has instead stressed the importance of leadership^41 45 54 73^ and suggested adapting leadership styles to address the particularities of the distributed setting and to enhance effectiveness and performance.^4041 43^,^53^

Over the past 20 years, significant advancements have been made in the communication practices of distributed teams. Early challenges such as potential breakdowns, mistrust, conflicts and power struggles^34 66 74 75^ have been mitigated by improvements in communication technologies like high-quality videoconferencing. These technologies now facilitate richer interactions and better integration of social cues. Additionally, strategies have come to be focused on promoting balanced participation and inclusivity, ensuring all team members can effectively contribute.^59 60 69 72^ As a result, more recent research has found no significant difference in communication quality between distributed and co-located teams.^3637 57 76^,^78^

Early studies exploring collaboration in distributed teams found that coordination delays were a barrier to successful performance, while more recent publications did not identify these problems to the same extent. The majority of studies discussing the importance and challenges of familiarity, trust and shared mental models in distributed teams were published in the early 2000s.^4349 61^,^64^ Recent studies suggest that technology may not necessarily affect teamwork processes,^41 70 77^ indicating that collaborations in distributed teams do not make teams work better or worse.^76^ Overall, the progression in understanding and managing collaboration over the past 20 years highlights the importance of adopting appropriate technological solutions and strategies to foster effective teamwork in distributed settings.

### Knowledge gaps in the research field

Leadership was highlighted as essential to the success of distributed teams, along with the need for the leader to have specialist training.^4042 47 62 63 79^,^81^ However, research on the effectiveness of different leadership training programmes for distributed teams in healthcare is notably lacking. Further, the factors influencing communication quality within distributed teams are still unclear, and analyses of various communication technologies do not consistently yield uniform outcomes,^3436 37 57 66 74^,^78^ suggesting further investigation is needed. Short-term distributed teams encounter unique challenges, such as a lack of familiarity and difficulty in building trust and a shared mental model.^4349 59 61^,^65^ These issues are distinct from established teams; however, there is only limited research addressing these specific disadvantages, particularly in healthcare settings where ad hoc situations, task urgency and patient demands add extra layers of complexity.

### Applications to healthcare

The articles underscored the importance of leadership training, given the unique challenges of working in a distributed manner.^4042 47 62 63 79^,^81^ In healthcare settings, in particular, leaders require training in managing task changes, navigating complexity, fostering collaboration and effectively using this technology while supporting team members in its usage.^40 42 82^ Additionally, ensuring that isolated team members feel included, involved and part of the team is crucial for leaders in the distributed team.^69 72^ Some studies suggested that richer technology, such as videoconferencing, would be preferable for distributed teams,^26 67 74 75^ particularly for complex tasks, as it enables social cues and leads to better intrateam communication and performance.^54 80 83 84^ Other research has highlighted the importance of selecting communication technology that aligns with the team’s specific needs, as it can impact team effectiveness and communication.^53 80 85 86^ Furthermore, one study indicated that adopting an approach following the ‘swift trust model’, which assumes that trust is present from the start of the collaboration, can be effective for distributed teams.^61^ It has been reported that when trust and a shared mental model are present, distributed teams can achieve outcomes comparable to co-located teams.^43 68^

## Discussion

The aim of this ScR was to map knowledge on leadership, communication and collaboration in short-term distributed teams across various fields. By understanding the dynamics of distributed teamwork in diverse contexts, we can potentially transfer and adapt fundamental knowledge and best practices to healthcare. This could involve optimising the organisation, developing educational and training programmes, and proposing future research. Ultimately, the success of distributed healthcare teams in their tasks could lead to enhanced patient outcomes.^19^

Regarding identified patterns, the findings suggest that critical aspects include clear communication, shared mental models and trust supported by effective leadership.^18^,^22^ These findings align with existing literature on co-located teams,^24^ emphasising the importance of these factors for team performance. However, reduced interaction and decreased social cues make these challenges more demanding for distributed teams.^26 52 60 67^ Therefore, using approaches that promote non-verbal communication should be beneficial for distributed healthcare teams. For instance, telemedicine has been found to improve communication and patient care by facilitating better visual interactions.^87^ Positioning technology strategically in the room could enhance non-verbal communication.

It was also suggested that using richer and more advanced technology, which allows clearer communication and the reading of non-verbal cues, could be beneficial.^26 72 80 83^ Although the use of synchronous visual technologies is increasing in healthcare,^40^ telephone consultations are still standard.^88^ In healthcare, however, seeing each other clearly may help prevent communication errors, as a lack of visual contact can contribute to mistakes.^89^ Clear communication is crucial in delivering safe, high-quality patient care^19^ and investing in richer technology that enables visual communication with patients and teams could therefore be advantageous.

The practical implications revealed by this ScR emphasise several aspects. In distributed teams, the team size and the location of members can vary.^35^ Considering that some members of healthcare teams may be with the patient while others are elsewhere, it becomes crucial to clearly define who is responsible for what and what role each team member plays in this collaboration. In healthcare, interprofessional teams often work in ad hoc teams. These teams have an asymmetry in knowledge,^40^ as each profession contributes different expertise to the team.^19 90^ Those on-site perform practical tasks, whereas remote members do not need that capability but instead may contribute knowledge, leadership and situational awareness. Effective leadership training is therefore essential.^69 72^ Even though research on this is lacking, it seems important for leadership training to focus on including all team members, clear communication and effectively using this technology.^40 42 49 79^

Nursing and medical students today frequently practice simulation and teamwork during their education. However, to our knowledge, there is no training in the workplace or during educational programmes that specifically prepares them for this type of collaboration in the distributed setting. It is essential that leaders and all team members understand how to facilitate visibility, read each other’s non-verbal cues, ensure everyone is included in the team, clarify and define the roles and responsibilities of themselves and others and use the technology effectively. Training programmes should be developed to address these specific skills, ensuring that all members of distributed healthcare teams are well-prepared to collaborate effectively.

The work environment in healthcare, especially during emergencies, is often characterised by high-stress levels, heavy workloads, complexity and significant consequences for decision-making and errors in actions.^91^ Studies examining teamwork in distributed teams in other contexts did not adequately consider these factors, which need to be examined in future research.

Over the past 20 years, considerable advances have been made in the literature, shifting focus from early 21st-century difficulties to providing more solutions. Technological advances and their impact during this period should be considered when interpreting the results. Additionally, societal changes have led to a population that is generally more adept at handling technology. The increased familiarity with and reliance on technology in everyday life has further facilitated its integration into various professional fields, including healthcare.^92^ Several international organisations, such as the WHO^93^ and the American Telemedicine Association,^94^ emphasise the importance of integrating advanced technologies to enhance healthcare delivery and patient outcomes. According to the WHO, telemedicine and other digital health solutions are vital for addressing healthcare disparities, particularly in remote and underserved areas, where approximately 2 billion people lack access to essential health services due to healthcare professionals being concentrated in urban areas.^95^ The COVID-19 pandemic accelerated the adoption of telehealth technologies. Many healthcare systems rapidly integrated these technologies to maintain continuity of care while minimising the risk of virus transmission.^9 10^

Despite the understanding that distributed teams are essential for providing equitable care and today’s technical advancement, several important research gaps remain in the literature, particularly in healthcare. This ScR found only nine articles concerning distributed healthcare teams, highlighting the lack of research in this context. However, our latest data searches showed a growing proportion of healthcare research between 2023 and 2024, which may indicate a trend towards increasing prevalence. Although they did not meet our inclusion criteria, many studies related to the pandemic demonstrate how healthcare has had to rely more on technology to gather teams.

## Strengths and limitations

This study provides a comprehensive and systematic overview of research on leadership, communication and collaboration in the field of short-term distributed teams using synchronised audio-visual technology. A notable strength of this study is that we updated our search twice to include the most recent and relevant studies. We have presented the evidence reported from various fields and identified a lack of research in healthcare. However, the broad scope of the study made it challenging to identify key factors specific to healthcare, as some articles did not provide enough details. Including research from different types of teams may have generated contradictory results. Additionally, as we did not place any restrictions on publication date, the reviewed studies covered a long period during which technological advances have significantly impacted distributed teamwork.

Since quality appraisal was not included in the methodology, the quality of the included articles has not been determined.^30^ We included conference papers, systematic reviews, theses and book chapters in accordance with the method described by Arksey and O’Malley.^30^ Including conference papers can improve the comprehensiveness of a review, but the information may not always be reliable or complete.^96^ Book chapters tend to generalise outcomes, making it difficult to extract data relevant to distributed healthcare teams. To improve reporting, we used the PRISMA-ScR and PAGER frameworks.^33^

## Conclusion

Effective leadership is crucial in addressing the specific challenges of distributed teams. Clear communication and appropriate use of technology are essential to overcome delays and misunderstandings. Collaboration is influenced by factors such as team familiarity and trust, which are harder to establish in distributed settings. Additionally, more empirical research is needed to understand distributed healthcare settings and develop effective training strategies. Future research should focus on developing and evaluating tailored training programmes to enhance leadership, communication and collaboration in these teams. This is particularly important in healthcare, where teams operate under time constraints and high patient care demands.

## supplementary material

10.1136/bmjopen-2023-081878 online supplemental file 1

10.1136/bmjopen-2023-081878 online supplemental file 2

10.1136/bmjopen-2023-081878 online supplemental file 3

10.1136/bmjopen-2023-081878 online supplemental file 4

## Data Availability

Data are available on reasonable request.
