# Supplementary material for: Mapping leadership, communication and collaboration in short-term distributed teams across various contexts: a scoping review
Source: BMJ Open. 2024 Oct 23;14(10):e081878. doi: 10.1136/bmjopen-2023-081878 (PMC11499798; doi:10.1136/bmjopen-2023-081878)
Supplement: online supplemental file 2 [file bmjopen-14-10-s002.pdf]

Initial database search: May 2021

**PubMed**	(‘distributed team*’[tiab] OR ‘virtual team*’[tiab] OR virtuality[tiab] OR ‘ad hoc team*’[tiab]) AND (teamwork[tiab] OR leadership[mesh] OR leadership[tiab] OR communication[mesh] OR communication[tiab] OR collaboration[tiab] OR cooperation[tiab] OR coordination[tiab])
**CINAHL**	(TI (‘distributed team*’ OR ‘virtual team*’ OR virtuality OR ‘ad hoc team*’) OR AB (‘distributed team*’ OR ‘virtual team*’ OR virtuality OR ‘ad hoc team*’)) AND (TI (teamwork OR leadership OR communication OR collaboration OR cooperation OR coordination) OR AB (teamwork OR leadership OR communication OR collaboration OR cooperation OR coordination) OR MH ‘communication+’ OR SU (communication OR leadership))
**APA PsycINFO**	(DE ‘virtual teams’ OR TI (‘distributed team*’ OR ‘virtual team*’ OR virtuality OR ‘ad hoc team*’) OR AB (‘distributed team*’ OR ‘virtual team*’ OR virtuality OR ‘ad hoc team*’) OR KW (‘distributed team*’ OR ‘virtual team*’ OR virtuality OR ‘ad hoc team*’)) AND (DE (teamwork OR collaboration OR cooperation OR ‘electronic collaboration’) OR MH (leadership OR communication) OR TI (teamwork OR leadership OR communication OR collaboration OR cooperation OR coordination) OR AB (teamwork OR leadership OR communication OR collaboration OR cooperation OR coordination) OR KW (teamwork OR leadership OR communication OR collaboration OR cooperation OR coordination))
**Scopus**	TITLE-ABS-KEY(‘distributed team*’ OR ‘virtual team*’ OR virtuality OR ‘ad hoc team*’) AND TITLE-ABS-KEY(teamwork OR leadership OR communication OR collaboration OR cooperation OR coordination)

Updated database search: February 2023

**PubMed**	
#1	(‘distributed team*’[tiab] OR ‘virtual team*’[tiab] OR virtuality[tiab] OR ‘ad hoc team*’[tiab])
#2	(teamwork[tiab] OR leadership[mesh] OR leadership[tiab] OR communication[mesh] OR communication[tiab] OR collaboration[tiab] OR cooperation[tiab] OR coordination[tiab])
#3	#1 AND #2
#4	2021/05/01:2025/05/01[crdt]
#5	#3 AND #4
**CINAHL**	
S1	(TI (‘distributed team*’ OR ‘virtual team*’ OR virtuality OR ‘ad hoc team*’) OR AB (‘distributed team*’ OR ‘virtual team*’ OR virtuality OR ‘ad hoc team*’))
S2	(TI (teamwork OR leadership OR communication OR collaboration OR cooperation OR coordination) OR AB (teamwork OR leadership OR communication OR collaboration OR cooperation OR coordination) OR MH ‘communication+’ OR SU (communication OR leadership))
S3	S1 AND S2
S4	EM 20210501-20251231
S5	S3 AND S4
**APA PsycINFO**	
S1	(DE ‘virtual teams’ OR TI (‘distributed team*’ OR ‘virtual team*’ OR virtuality OR ‘ad hoc team*’) OR AB (‘distributed team*’ OR ‘virtual team*’ OR virtuality OR ‘ad hoc team*’) OR KW (‘distributed team*’ OR ‘virtual team*’ OR virtuality OR ‘ad hoc team*’)) AND (DE (teamwork OR collaboration OR cooperation OR ‘electronic collaboration’) OR MH (leadership OR communication) OR TI (teamwork OR leadership OR communication OR

	collaboration OR cooperation OR coordination) OR AB (teamwork OR leadership OR communication OR collaboration OR cooperation OR coordination) OR KW (teamwork OR leadership OR communication OR collaboration OR cooperation OR coordination))
S2	(DE (‘electronic collaboration’ OR ‘electronic communication’ OR ‘computer mediated communication’)) AND (TI team* OR AB team* OR KW team*)
S3	S1 OR S2
S4	RD 20210501-20251231
S5	S3 AND S4
S6	Publication Year: 2021–2023
**SCOPUS**	
1	TITLE-ABS-KEY(‘distributed team*’ OR ‘virtual team*’ OR virtuality OR ‘ad hoc team*’)
2	TITLE-ABS-KEY(teamwork OR leadership OR communication OR collaboration OR cooperation OR coordination)
3	#1 AND #2
4	PUBYEAR AFT 2020
5	#3 AND #4

Updated database search: May 2024

APA PsycINFO via EBSCOHost 240523. Boolean/Phrase. No filter.

#	Search string	Hits
S1	(DE “virtual teams” OR TI (”distributed team*” OR “virtual team*” OR virtuality OR “ad hoc team*”) OR AB (”distributed team*” OR “virtual team*” OR virtuality OR “ad hoc team*”) OR KW (”distributed team*” OR “virtual team*” OR virtuality OR “ad hoc team*”)) **AND** (DE (teamwork OR collaboration OR cooperation OR “electronic collaboration”) OR MH (leadership OR communication) OR TI (teamwork OR leadership OR communication OR collaboration OR cooperation OR coordination) OR AB (teamwork OR leadership OR communication OR collaboration OR cooperation OR coordination) OR KW (teamwork OR leadership OR communication OR collaboration OR cooperation OR coordination))	1185
S2	(DE (”electronic collaboration” OR “electronic communication” OR “computer mediated communication”)) AND (TI team* OR AB team* OR KW team*)	473
S3	S1 OR S2	1506
S4	RD 20230101-20251231	276007
S5	S3 AND S4	94
S6	Publication Year: 2021-2024	94

PubMed 20240523. No filter

#	Search string	Hits
#1	(”distributed team*”[tiab] OR “virtual team*”[tiab] OR virtuality[tiab] OR “ad hoc team*”[tiab])	412
#2	(teamwork[tiab] OR leadership[mesh] OR leadership[tiab] OR communication[mesh] OR communication[tiab] OR collaboration[tiab] OR cooperation[tiab] OR coordination[tiab])	1007119

#3	#1 AND #2	208
#4	2023/03/01:2025/05/01[crdt]	1909857
#5	#3 AND #4	30

CINAHL via EBSCOHost 240523. Boolean/Phrase. No filter

#	Search string	Hits
S1	(TI (”distributed team*” OR “virtual team*” OR virtuality OR “ad hoc team*”) OR AB (”distributed team*” OR “virtual team*” OR virtuality OR “ad hoc team*”))	208
S2	(TI (teamwork OR leadership OR communication OR collaboration OR cooperation OR coordination) OR AB (teamwork OR leadership OR communication OR collaboration OR cooperation OR coordination) OR MH “communication+” OR SU (communication OR leadership))	575319
S3	S1 AND S2	135
S4	EM 20230301-20251231	305468
S5	S3 AND S4	13

Scopus 240523. No filter.

#	Search string	Hits
1	TITLE-ABS-KEY("distributed team*" OR "virtual team*" OR virtuality OR “ad hoc team*”)	10201
2	TITLE-ABS-KEY(teamwork OR leadership OR communication OR collaboration OR cooperation OR coordination)	4059940
3	#1 AND #2	4829
4	PUBYEAR AFT 2022	5755500
5	#3 AND #4	356
