# Supplementary material for: Mapping leadership, communication and collaboration in short-term distributed teams across various contexts: a scoping review
Source: BMJ Open. 2024 Oct 23;14(10):e081878. doi: 10.1136/bmjopen-2023-081878 (PMC11499798; doi:10.1136/bmjopen-2023-081878)
Supplement: online supplemental file 3 [file bmjopen-14-10-s003.pdf]

Supplementary material 2

**Patterns**	**Advances**	**Gaps**	**Evidence for practice**	**Research recommendations**
Leadership in distributed teams<br><br>The role of inclusive leadership in mitigating exclusion within distributed teams, where frequent contributors often emerge as leaders.	*Earlier studies* highlighted challenges in leadership for distributed teams.<br>*Recent research* explores various leadership styles and strategies for effective leadership.	Research on the effectiveness of different leadership training programs for distributed teams is notably lacking.	Training programs for leaders address the unique aspects of the distributed setting, including leadership skills and technology proficiency.	Research is needed on the leadership challenges in healthcare's distributed teams, particularly those related to managing urgent tasks while upholding patient care standards.
Communication in distributed teams<br><br>Clear communication is important and a balance between sites must be achieved.	*Earlier studies* suggested that distributed teams face communication issues.<br>*Recent research* shows no significant difference in communication quality.	There is a lack of clarity regarding the factors that influence communication quality within distributed teams.	Richer technology is recommended, as it enables social cues. This is particularly important when dealing with urgent and complex tasks.	Research on communication strategies in short-term distributed teams and different communication technologies is needed, as studies have shown inconsistent results.
Collaboration in distributed teams<br><br>Short-term teams face challenges in building familiarity and trust compared to experienced ongoing teams.	*Earlier studies* addressed the impact of missing nonverbal cues on team awareness.<br>*Recent research* indicates technology has no significant effect on collaboration.	There is a paucity of research on how to address the disadvantages faced by short-term distributed teams.	For short-term teams, the “swift trust model” recommends acting as if trust is already present from the start of the collaboration. This approach helps build trust quickly and achieve outcomes.	Research is needed on effective approaches for fostering trust and creating a shared understanding among short-term distributed teams.
