# Supplementary material for: Mapping leadership, communication and collaboration in short-term distributed teams across various contexts: a scoping review
Source: BMJ Open. 2024 Oct 23;14(10):e081878. doi: 10.1136/bmjopen-2023-081878 (PMC11499798; doi:10.1136/bmjopen-2023-081878)
Supplement: online supplemental file 4 [file bmjopen-14-10-s004.pdf]

Characteristics of the included articles in alphabetical order.

Author & year	Title	Country	Article type	Design	Setting	Objective	Terms
Alhawary, 2012	Factors affecting virtual teamwork collaboration: a case study in the Jordanian Royal Medical Services	Asia	Journal article	Quantitative empirical	Healthcare	Examine the effects of factors such as trust, cultural differences, time differences, and language on a virtual team's work collaboration	Virtual teams
Anderson et al., 2007	Virtual team meetings: an analysis of communication and context	Europe	Journal article	Mixed-method empirical	Industrial & engineering	Investigate the amount and patterns of interaction among team members and the content of the discussions	Virtual teams
Avolio et al., 2001	Virtual teams: implications for e-leadership and team development	USA	Book chapter		Non-specific	Examine how trust contributes to the development and performance of virtual teams	Virtual team, virtual context dispersed location
Baker, 2019	Communication and trust in virtual and face-to-face teams	USA	Thesis	Quantitative experimental	University, aeronautical	Evaluate the constructs of trust, communication, and effectiveness in virtual teams	Virtual team
Bell & Kozlowski, 2002	A typology of virtual teams: implications for effective leadership	USA	Journal article		Non-specific	Develop a theoretical framework to improve understanding of virtual teams and identify implications for effective leadership	Virtual teams
Bennett, 2010	Development and performance of distributed teams: examining differences between asynchronous and synchronous communication in planning task execution	USA	Thesis	Quantitative experimental	Military, aircrew	Compare distributed computer-mediated teams who chose different communication methods in terms of team development and performance	Virtual teams, distributed teams
Bolle et al., 2009	Video conferencing versus telephone calls for teamwork across hospitals: a qualitative study on simulated emergencies	Europe	Journal article	Qualitative empirical	Healthcare	Study whether videoconferencing could improve communication and team function between rural and central emergency hospital teams with several participants at either side of the video link	Virtual teams

Bos et al., 2002	Effects of four computer-mediated communication channels on trust development	USA	Conference paper	Quantitative	University, game-playing	Examine trust development in a set of four media conditions	Virtual teams
Brown et al., 2021	Leadership and virtual team performance: a meta-analytic investigation	USA	Journal article	Quantitative	Non-specific	Examine existing empirical evidence regarding the effect of leadership on virtual team performance.	Virtual teams
Butler et al., 2019	The impact of telemedicine on teamwork and workload in pediatric resuscitation: a simulation-based, randomised controlled study	USA	Journal article	Quantitative empirical	Healthcare	Evaluate the impact of telemedicine technology on care delivered during a simulated pediatric resuscitation	Telepresent providers
Driskell et al., 2003	Virtual teams: effects of technological mediation on team performance	USA	Journal article		Non-specific	Examine the effects of technological mediation on team processes such as cohesiveness, status and authority relations, counter-normative behaviour, and communication	Virtual teams
Driskell & Salas, 2006	Groupware, group dynamics, and team performance	USA	Book chapter		Non-specific	Address challenges in supporting collaborative activities in distributed teams	Virtual teams, distributed teams
Eseryel et al., 2021	Functional and visionary leadership in self-managing virtual teams	USA	Journal article		Non-specific	Present a theory of leadership in self-managing virtual teams	Self-managing virtual teams
Fang et al., 2014	Real-time video communication improves provider performance in a simulated neonatal resuscitation	USA	Journal article	Quantitative empirical	Healthcare	Determine if a real-time audio-visual link with a neonatologist, termed video-assisted resuscitation or VAR, improves provider performance during a simulated neonatal resuscitation scenario	Video-assisted resuscitation (VAR)
Fiore et al., 2003	Distributed coordination space: toward a theory of distributed team process and performance	USA	Journal article		Non-specific	Present a framework of “distributed coordination” for developing the most appropriate principles for distributed team performance	Distributed teams

Gibbs et al., 2017	Investigating the impacts of team type and design on virtual team processes	USA	Review	Empirical	Non-specific	Illustrate how study design impacts our knowledge of virtual teams; focus on three key research topics of leadership, cultural composition, and technology use; and analyse how findings on these topics are shaped by team type, configuration and study design	Virtual teams
Gilson et al. 2015	Virtual teams research: 10 years, 10 themes, and 10 opportunities	USA	Review		Non-specific	Review the last 15 years of research on virtual teams, team type, and study design	Virtual teams
Guo et al., 2006	Effectiveness of meeting outcomes in virtual vs. face-to-face teams: a comparison study in China	Asia	Conference paper	Quantitative experimental	Business	Examine how virtual teams interacting via videoconferencing systems may enhance their team outcomes	Virtual teams
Guo et al., 2009	Improving the effectiveness of virtual teams: a comparison of video-conferencing and face-to-face communication in China	Asia	Journal article	Quantitative experimental	Business	Examine the impact of dialogue technique on virtual team relational development and meeting outcomes by comparing team interactions in traditional face-to-face teams and teams interacting via videoconferencing systems	Virtual teams
Hambley et al., 2007	Virtual team leadership: the effects of leadership style and communication medium on team interaction styles and outcomes	Canada	Journal article	Quantitative experimental	Non-specific	Examine how leadership influences team cohesion as an outcome in its own right	Virtual teams
Hassell, 2016	The message is more than the medium: a study of media theories and impacts	USA	Conference paper	Quantitative experimental	Business	Provide clarity on media impact theories and better understand media impacts and team issues	Virtual teams
Hassell & Limayem, 2020	Media impacts and performance in dispersed teams	USA	Journal article	Quantitative experimental	Business	Provide additional clarity and insights on media impact theories and issues to enhance organisations’ use of dispersed teams	Dispersed teams

Hayward, 2011	Shared mental model development during technology-mediated collaboration	USA	Journal article	Quantitative experimental	Software design	Examine how collaboration mode (face-to-face vs. videoconferencing technology-mediated virtual teams) shapes the negotiated shared interpretation of ideas needed for shared mental model construction	Virtual teams
Hayward, 2012	Technology-mediated collaboration shared mental model and task performance	USA	Journal article	Mixed-method experimental	Software design	Examine how the shared mental model mediates the impact of collaboration mode on team productivity and process satisfaction	Virtual teams
Henrie & D'Antonio, 2016	Leading from a Distance: An Exploratory Systematic Review	USA	Conference paper			Provide insight and guidance to practitioners faced with virtual team leadership challenges	Virtual teams, geographically distributed teams, dispersed teams
Heuser, 2010	An examination of the use of synchronous computer-mediated communication technology in work teams	USA	Thesis	Quantitative experimental	Business	Explore the impact of member structural arrangement across the virtual space on critical team processes and team effectiveness outcomes when communications technology richness varies	Virtual teams
Hoyt, 2013	Leadership within virtual contexts	Europe	Book chapter	Empirical	Non-specific	Provide an overview of leadership within virtual contexts by focusing on relevant theories and empirical research	Virtual teams
Hughes et al., 2021	Trauma, teams, and telemedicine: evaluating telemedicine and team-work in a mass casualty simulation	USA	Journal article	Quantitative experimental	U.S. Army surgical teams	Investigate the utility of a telemedical device to a geographically dispersed team	Geographically dispersed team
Kahai et al., 2017	E-leadership	USA	Book chapter		Non-specific	Discuss the various themes pertaining to e-leadership in the literature	Virtual teams, e-leadership

Keijser et al., 2016	Physician leadership in e-health? A systematic literature review	Europe	Review		Healthcare	Systematically review the literature on the roles of physicians in virtual teams delivering healthcare for effective "physician e-leadership" and implementation of e-health	Virtual teams Physician e-leadership
Kennel, et al., 2021	Virtual teamwork in Healthcare delivery: I-O Psychology in telehealth research and Practice	USA	Journal article		Healthcare	Discuss how the science of teams and virtual teams can guide efforts to create and develop effective virtual healthcare teams and identify opportunities where research on virtual healthcare teams can advance the science of the field	Virtual healthcare teams
Lacerenza et al., 2015	Team training for global virtual teams: strategies for success	USA	Book chapter		Non-specific	Identify the barriers to effective teamwork in global virtual teams and outline team training strategies that may help mitigate the loss to team process and performance	Global virtual teams
Lazzara et al., 2015	Utilising telemedicine in the trauma intensive care unit: does it impact teamwork?	USA	Journal article	Quantitative experimental	Healthcare	Examine the impact of a telemedical robot on trauma intensive care unit clinician teamwork (i.e. team attitudes, behaviours, and cognitions) during patient rounds	Remote rounds with telemedicine
Ligda et al., 2015	Effectiveness of advanced collaboration tools on crew communication in reduced crew operations	USA	Conference paper	Quantitative empirical	Airline industry	Examine operational performance and verbal communication in airline flight crews under reduced crew operations	Distributed teams
Linebarger et al., 2005	Benefits of synchronous collaboration support for an application-centered analysis team working on complex problems: a case study	USA	Conference paper	Mixed-method experimental	Agent-based laboratory economics	Examine whether synchronous collaboration capability through a particular application improved the ability of the team to form a common mental model of the analysis problem(s) and solution(s)	Computer-mediated collaboration
Maynard et al., 2017	Virtual teams	USA	Book chapter		Non-specific	Provide an overview of team virtuality	Virtual teams

Miloslavic et al., 2015	Structuring successful global virtual teams	USA	Book chapter		Non-specific	Summarise the theoretical and empirical research on global teams and synthesise useful recommendations for organisations seeking to compose global teams	Global virtual teams
Peñarroja et al., 2013	The effects of virtuality level on task-related collaborative behaviours: the mediating role of team trust	Europe	Journal article	Quantitative experimental	Psychology, university	Analyse the mediating role of team trust in the relationship between virtuality level and task-related collaborative behaviours	Virtual teams
Priest et al., 2006	Virtual teams: creating context for distributed teamwork	USA	Book chapter		Non-specific	Present five areas of focus that address aspects of virtual teamwork	Virtual teams, distributed teamwork
Rennie, et al., 2023	Communication style drives emergent leadership attribution in virtual teams	USA	Journal article	Quantitative experimental	University, fraternity	Investigate how pre-assigned leadership roles within a professional network influence the attribution of emergent leadership during virtual group interactions	Virtual teams
Rockwood & Nathan-Robert, 2018	A systematic review of communication in distributed crews in high-risk environments	USA	Conference paper		Aerospace, aviation domains	Examine communication in distributed teams in high-risk environments to provide an overview of the literature	Distributed teams
Roy, 2013	Virtual prowess: The keys to effective virtual leadership	USA	Book chapter		Non-specific	Discuss keys to effective virtual leadership	Virtual teams
Saunders & Ahuja, 2006	Are all distributed teams the same? Differentiating between temporary and ongoing distributed teams	USA	Journal article		Non-specific	Present a framework for understanding the differences between temporary and ongoing distributed teams' structure, processes, and outcomes	Distributed teams, virtual teams
Schmidtke & Cummings, 2017	The effects of virtualness on teamwork behavioural components: the role of shared mental models	USA	Review		Non-specific	Present the ways in which shared mental models affect the behavioural components of teamwork within virtual teams	Virtual teams

Begränsad delning

Shuffler et al., 2010	Leading one another across time and space: exploring shared leadership functions in virtual teams	USA	Journal article		Non-specific	Provide a framework for specific leader functions that members of virtual teams may share	Virtual teams
Shuffler et al., 2016	#TeamLeadership: Leadership for today's multicultural, virtual, and distributed teams	USA	Book chapter		Non-specific	Explore existing research for an improved understanding of how to best utilise collective team leadership as a mechanism for effectively working in today's multicultural, distributed, and virtual environments	Virtual teams Distributed teams
Tripathy, et al., 2023	Virtual Teams: Analysis of best technology to optimise with the goal setting of all technical aspects	Asia	Conference paper		Non-specific	Review the current literature on managing virtual teams and summarise best practices for effective remote team leadership and communication	Virtual teams
Tyran & Tyran, 2007	The role of leadership in virtual teams	USA	Book chapter		Non-specific	Provide a summary of the role of leadership in virtual teams and identify how a leader may influence a virtual team	Virtual teams
Van der Kleij et al., 2009	How conversations change over time in face-to-face and video-mediated communication	Europe	Journal article	Experimental	University, intellectual task	Investigate how communication, satisfaction, and task performance differ as a function of the group's communication environment, whether distributed or collocated and how these variables change over time	Virtual teams
Weisband, 2008	Research challenges for studying leadership at a distance	USA	Book chapter		Non-specific	Provide a perspective on leadership at a distance from the research that considers the role of single leaders with special traits and attributes to a more nuanced role of leadership emergence, technical expertise, and new authority structures in large-scale collaborations	Virtual teams

Weisband, 2008	Lessons about leadership at a distance and future research directions	USA	Book chapter		Non-specific	Provide a lens for future research	Geographically distributed communities, geographically dispersed teams, distant leadership
White et al., 2020	Ad hoc teams and telemedicine during COVID-19	USA	Journal article		Healthcare	Outline key aspects, development, and challenges faced by ad-hoc teams and how they were successful in the uncharted waters of a COVID-19 response	Geographically dispersed ad hoc teams
Xiao & Wei, 2008	A study on virtual team communication technologies and their applicabilities	Asia	Conference paper		Non-specific	Present a taxonomy of virtual team communication synchronisation, geographical distribution, and the number of senders	Virtual teams
Xiao et al., 2008	Adaptation of team communication patterns: exploring the effects of leadership at a distance, task urgency, and shared team experience	USA	Book chapter	Mixed-method experimental	Healthcare	Examine the hierarchy of leadership and team communication patterns within trauma resuscitation teams under varying conditions: (a) when the team leader was distant versus collocated, (b) when the team's task – patient treatment – was high versus low in urgency, and (c) when team members had more or less shared experience as a team	Distant leadership
Zaccaro & Bader, 2003	E-leadership and the challenges of leading e-teams: minimising the bad and maximising the good	USA	Journal article		Business	Discuss the challenges for e-leadership	E-teams
