# Supplementary material for: Mapping leadership, communication and collaboration in short-term distributed teams across various contexts: a scoping review
Source: BMJ Open. 2024 Oct 23;14(10):e081878. doi: 10.1136/bmjopen-2023-081878 (PMC11499798; doi:10.1136/bmjopen-2023-081878)
Supplement: Supplementary file 5 [file bmjopen-2023-081878.reviewer_comments.pdf]

PEER REVIEW HISTORY

BMJ Open publishes all reviews undertaken for accepted manuscripts. Reviewers are asked to complete a checklist review form and are provided with free text boxes to elaborate on their assessment. These free text comments are reproduced below.

ARTICLE DETAILS

Title (Provisional)

Mapping leadership, communication, and collaboration in short-term distributed teams across various contexts: a scoping review

Authors

Morian, Hanna Rebecka; Creutzfeldt, Johan; Hultin, Magnus; Härgestam, Maria

---

VERSION 1 - REVIEW

**Reviewer**	**1**
**Name**	**Jacobsen , Dag Ingvar**
**Affiliation**	**University of Agder, POlitical science and management**
**Date**	**30-Nov-2023**
**COI**	**none**

Comments on «Exploring teamwork….» bmjopen 2023 081878

This is an exploratory or scoping systematic review of a topic of increasing importance for most organizations, professions, and disciplines: teamwork in distributed teams. It is of special interest that the review focuses on teams working together for a short time (< 24h). The review follows established standards and guidelines in a very solid way, and there is little ground for being suspicious of the findings or the main conclusions.

I have one main comment, and a few minor ones.

Main comment:

This is a paper meant for publication in a medical journal. As such, it is of main interest to the reader to gain knowledge about working in short term teams within the health profession (i.e., hospitals and related situations). Although the authors state that there are not many studies in this area (9), I think these studies should be given some more space and be used to compare with the other 44 studies. First, I would appreciate is a short description of the 9 healthcare studies in the article; the situation(s) studied, the number of units (n) in each study, national context, and year. Now I must look in the appendix to find (some of) this. Second, the section called "discussion" is an ad-hoc summary of the studies on distributed team within healthcare. This is interesting, but I would also appreciate an

evaluation from the authors on any similarities and differences between the 9 healthcare studies and the rest. It may not be any differences across disciplines, but it would be interesting to see the authors' reflections on this.

Minor comments:

• "Short term" should be included in the title "Exploring teamwork within in short term distributed teams".

• On p. 3 I'm not sure whether the term "are especially effective" should be used. It is probably more correct to say that distributed teams are more often employed when facing emergencies. Whether they are effective or not is not easy to say.

As noted by the authors, the search is wide, and the scope is very broad. I just wonder how many "hits" the search would have provided if search words like communication, leadership, etc had been excluded, and the search had only focused on distributed teams and the like. Are there many studies of distributed teams not including communication, etc.? This would have been interesting to know.

On a final note, I would just like to thank the authors for putting so much work into this. Such studies are of great value.

**Reviewer**	**2**
**Name**	**Mastalerz, Katarzyna**
**Affiliation**	**University of Colorado School of Medicine**
**Date**	**14-Mar-2024**
**COI**	**NA**

Thank you for conducting this scoping review of distributed team with respect to leadership, communication and collaboration. It is important to understand what is already known about distributed teams in order to improve communication and collaboration in distributed healthcare teams. Good methods section. Clearly defined research question.

Major issues:

Results - you state that the reported data was guided by the 5 domains of the PAGER framework but the results are organized by categories that are named leadership, communication, and collaboration. Is confusing how you arrived from the PAGER framework to these outcome themes and I would either describe this in the methods, provide additional tables or graphs to clarify how these concepts were synthesized, and organize the results data with different categories.

Discussion - this seems to bring in significant new data that I think should be included in the results section. Whatever you found as a result of the scoping review should be reported as results whereas the discussion should summarize the data, nestle it within recent literature, provide significance, and future direction. I recommend rewriting the discussion section with that guidance after all the results are reported on in the results section. Statements such as "our scoping review showed that healthcare sector is increasingly utilizing technology to..." is a result that should be included in the correct section. No new information from the scoping review should be presented in the discussion. Also, please change redundant statements in the discussion (those that are identical to what you stated in results) to summaries.

Conclusion - it doesn't seem to emphasize the important aspects of what you found in the scoping review and I would change it to achieve this purpose.

Minor issues: (please see my attachment with comments and highlights for this section, including clarifying all terms in the abstract, defining and backing up what "traditional" healthcare teams are, and fixing some run-on sentences in the discussion, rewriting a few areas for clarity.

****The reviewer provided a marked copy with additional comments. Please contact the publisher for full details.**

**Reviewer**	**3**
**Name**	**Nørgaard, Birgitte**
**Affiliation**	**Institute of Public Health, University of Southern Denmark, Centre for Applied Health Services Research**
**Date**	**18-Apr-2024**
**COI**	**None**

Thank you for the opportunity to read this manuscript. Overall, I find the manuscript well-written and well-structured - i do however, have some suggestions for improvement.

Abstract: there are a few, yet important, deviations from the PRISMA-ScR guideline recommendations, and I recommend that the abstract is re-structured according to the guideline (e.g., include background information).

As the updated search was conducted more than 1 year before submission, I recommend that the search is updated.

Introduction: I really wonder why you include a sentence on teams in industries, business and the military when your review focus on healthcare? I acknowledge that these teams might have been a fact in industries, business and the military prior to healthcare but if this is your message, please clarify.

Aim: It remains unclear what is meant by "short period" - and why this is of particular interest.

Methods: My compliments for applying both the framework by Arksey and O'Malley and PRISMA-ScR - I would however, recommend that you apply PRISMA as the overall structure of the methods section - this would in my opinion increase the readability of the section.

You define what is meant by a short period - but it remains unclear and somehow arbitrary that you chose 24 hours. Please elaborate on this and provide references for your choice.

You state that only one reason for exclusion was used - please mention this reason in the sentence - just for clarity. According to the flowchart, multiple reasons for exclusion were used? this is a bit confusing...

Charting the data: you write "findings on relevant teamwork concepts from the articles" - what what was considered relevant data her? Please, elaborate on this for clarity.

Discussion: You mention here barriers and challenges but to my knowledge, this is the first time this is mentioned in the manuscript. Please, do not mention new concepts in the summary of evidence.

Strengths and limitations: You state that you have identified several research gaps - this might be true, but you haven't presented these clearly for the reader - please do so to make this sentence meaningful.

Flowchart: I find it unnecessarily confusing that you present the results from the two searches separately and I recommend that this is collapsed.

**Reviewer**	**4**
**Name**	**Kim, Se Young**
**Affiliation**	**Changwon National University**
**Date**	**24-Apr-2024**
**COI**	**No.**

I think this is a meaningful study as an examination of teamwork in distributed teams. This appears to be a study that did not directly identify the concept of teamwork, but instead investigated leadership, communication, and cooperation as key variables. If so, further description of the rationale for selecting these variables is necessary in the introduction. In the results, leadership, communication, and cooperation variables were described separately, but there is some overlap, so there is a need to revise them. The distinction between results and discussion is not clear, and the content of the discussion is relatively lacking, so additional description is needed. Thank you.

VERSION 1 - AUTHOR RESPONSE

Reviewer 1

Dr. Dag Ingvar Jacobsen, University of Agder

**Reviewer's comments**		**Authors' response**
**#1**	This is an exploratory or scoping systematic review of a topic of increasing importance for most organizations, professions, and disciplines: teamwork in distributed teams. It is of special interest that the review focuses on teams working together for a short time (< 24h). The review follows established standards and guidelines in a very solid way, and there is little ground for being suspicious of the findings or the main conclusions.<br>I have one main comment, and a few minor ones.	Thank you for your comments regarding the thoroughness and solidity of our ScR. We greatly appreciate your feedback.
**#2**	This is a paper meant for publication in a medical journal. As such, it is of main interest to the reader to gain knowledge about working in short term teams within the health profession (i.e., hospitals and related situations). Although the authors state that there are not many studies in this area (9), I think these studies should be given some more space and be used to compare with the other 44 studies.<br><br>First, I would appreciate is a short description of the 9 healthcare studies in the article; the situation(s) studied, the number of units (n) in each study, national context, and year. Now I must look in the appendix to find (some of) this.	Thank you for this suggestion, which has helped us to enhance the quality and depth of our article.<br><br>While our aim was to explore various contexts, we realise the importance of giving due attention to the unique dynamics of short-term teams within the healthcare profession.<br><br>We have added a description of the nine healthcare studies, detailing the specific situations studied, the national context, and the year of publication. The new text reads as follows (page 10, lines 217-227):<br><br>A subset of the included articles (n=9) focused specifically on healthcare settings. These articles were

	Second, the section called “discussion” is an ad-hoc summary of the studies on distributed team within healthcare. This is interesting, but I would also appreciate an evaluation from the authors on any similarities and differences between the 9 healthcare studies and the rest. It may not be any differences across disciplines, but it would be interesting to see the authors’ reflections on this.	published between 2008 and 2021 and exhibited some recurring themes and characteristics. Several articles focused on simulations or experiments involving healthcare professionals. For instance, Bolle et al.[30] and Butler et al.[31] conducted simulations with professionals from Norway and the USA, respectively, while Fang et al.[32] and Lazzara et al.[33] conducted randomised studies with US healthcare professionals. Xiao et al.[34] performed an experimental study to analyse remote leadership of real-life trauma resuscitation teams in a medical setting in the USA. Editorials by Kennel et al.[35] and White et al. 6 offered insights and reflections on the impact of distributed healthcare teams during the COVID-19 pandemic. Alhawary[28] explored virtual teamwork within the Jordanian Royal Medical Services, whereas Keijser et al.[37] reviewed current healthcare research.<br><br>We acknowledge the need for a revised discussion. The discussion now more explicitly focuses on healthcare, highlighting specific challenges and considerations for distributed healthcare teams. We hope this adequately addresses your point.
**#3**	“Short term” should be included in the title “Exploring teamwork within in short term distributed teams”.	We agree and have revised the title as follows:<br><br>Mapping leadership, communication, and collaboration in short-term distributed teams across various contexts: a scoping review
**#4**	On p. 3 I’m not sure whether the term “are especially effective” should be used. It is probably more correct to say that distributed teams are more often employed when facing emergencies. Whether they are effective or not is not easy to say.	You make a good point about avoiding the term “effective” in situations where effectiveness might not be definitively determined. We have revised the sentence accordingly to ensure clarity and accuracy, as follows (page 3, lines 72):<br><br>Distributed settings are additionally employed to organize the workforce when facing emergencies such as the COVID-19 pandemic, during which healthcare systems were forced to evolve and adjust.
**#5**	As noted by the authors, the search is wide, and the scope is very broad. I just wonder how	Thank you for this interesting question.

	many "hits" the search would have provided if search words like communication, leadership, etc had been excluded, and the search had only focused on distributed teams and the like. Are there many studies of distributed teams not including communication, etc.? This would have been interesting to know.	We would have encountered more studies on distributed or virtual teams if we had excluded aspects such as leadership, communication, and collaboration from our database search criteria since the search would have been broader. A test search in Scopus with only ("Distributed team*" OR "Virtual Team*") generated over 5900 articles, approximately 2000 more than our initial search. However, using such a broad search string meant that the search also retrieved studies with titles and abstracts that did not include the key aspects of leadership, communication, or collaboration. Considering that our interest lay explicitly in mapping these aspects of teamwork within distributed teams, we included them in our search in order to obtain a more relevant set of studies for our research question.
**#6**	On a final note, I would just like to thank the authors for putting so much work into this. Such studies are of great value.	Thank you for acknowledging our efforts. We genuinely appreciate your acknowledgement of the value of our study. Your feedback has been invaluable in enhancing the quality of our work.

Reviewer 2

Prof. Katarzyna Mastalerz, University of Colorado School of Medicine

**Reviewer's comments**		**Authors' response**
**#1**	Results - you state that the reported data was guided by the 5 domains of the PAGER framework but the results are organized by categories that are named leadership, communication, and collaboration. Is confusing how you arrived from the PAGER framework to these outcome themes and I would either describe this in the methods, provide additional tables or graphs to clarify how these concepts were synthesized, and organize the results data with different categories.	Thank you for your useful feedback, which will strengthen our article.<br><br>We acknowledge the confusion regarding the organization of our results based on the PAGER framework and are grateful to you for highlighting this issue. We have addressed this concern by adding a table (Supplementary Material 2) covering each aspect of the PAGER framework. We hope this clarification will enhance the understanding of our methodology and the synthesis of our results. The new table is also now mentioned in the text as follows (pages 7-8, lines 168-173):<br><br>Supplementary Material 2 provides a summary of our findings in terms of the PAGER domains: patterns of distributed leadership, communication, and collaboration (Patterns); advances made in the field (Advances); reflections on the paucity of research (Gaps); practical implications for

		distributed health professionals and organisations using distributed teams (Evidence for practice); and suggested areas for future research (Research recommendations).
**#2**	Discussion - this seems to bring in significant new data that I think should be included in the results section. Whatever you found as a result of the scoping review should be reported as results whereas the discussion should summarize the data, nestle it within recent literature, provide significance, and future direction. I recommend rewriting the discussion section with that guidance after all the results are reported on in the results section. Statements such as "our scoping review showed that healthcare sector is increasingly utilizing technology to..." is a result that should be included in the correct section. No new information from the scoping review should be presented in the discussion. Also, please change redundant statements in the discussion (those that are identical to what you stated in results) to summaries.	We have substantially revised this section in order to strengthen the article, focusing on what can be learned in healthcare from other contexts. We hope that this revision meets your expectations and addresses your feedback.
**#3**	Conclusion - it doesn't seem to emphasize the important aspects of what you found in the scoping review and I would change it to achieve this purpose.	Thank you for pointing this out. We have revised the conclusion to better align with the results, as follows (page 19, lines 453-461):
**#4**	Reviewer's comments embedded within the article:<br><br>• "Team Opacity" Define this or say what it is - it's a commonly understood term for the abstract	Below are responses to the reviewer's comments that were embedded within the article. We greatly appreciate these detailed comments, as they have enabled us to enhance the quality of the article.<br><br>We have clarified the meaning of "team opacity" as follows (page 2, lines 46–47): "Team opacity", the absence of nonverbal cues and reduced awareness of team members' actions, can occur in distributed teams relying on technology.

	• Is this the appropriate term here: disclosures?	We have replaced "disclosures" with following text; Developing shared mental models and trust is more challenging, leading to uncertainty and reduced information sharing (page 3, line 30).
	• In distributed care settings	We agree with and have now implemented the reviewer's deletion of the phrase "in distributed care settings" in the abstract.
	• What kind of technology OR used technology in contrast with..	We appreciate your interest in this aspect of our study. It is important to clarify that the definition of distributed teams does not assume specific technologies, but rather emphasises the use of technology. The focus of our study was not on specific technological solutions (as long as they were synchronous and visual) but rather on understanding remote leadership, communication, and collaboration. We have revised the sentence to instead mention "various technologies" (page 3, line 71).
	• Are these traditional? How? A lot of past healthcare teams are no co-located	The meaning of "traditional" depends on one's perspective. Traditionally, teams caring for patients in hospitals or primary care centres have been in the same physical location as the patient. Virtual work has increased in recent years, especially during the COVID-19 pandemic, thanks to technological advancements. For example, distributed teams have been implemented in rural areas of Sweden, which previously was not possible. Furthermore, the literature on teamwork, especially in emergency healthcare, often assumes that team members are physically present in the same location. Leadership roles are traditionally viewed from the perspective of an onsite leader.
	• Define map distributed?	We apologize for our previous phrasing, which was confusing. The aim has been revised to make it clear that "map" is being used as a verb, and we now use the phrase "to map knowledge". "Mapping" is a common term used to describe the approach in scoping reviews, referring to the process of systematically identifying existing knowledge on a specific topic.
	• What is Covidence?	Covidence is a software package similar to Rayyan for managing the phase of selecting articles. These tools help to streamline the

		process of screening and selecting studies for review. We now mention them earlier in the article, in the first sentence under "Selecting articles" and have added the word "software" to describe them.
	• "Initiating", is this the right word?	We have revised this sentence and removed the word "initiating" as follows: This could involve optimising the organisation, developing educational and training programs, and proposing future research (page 15, lines 355-356).
	• I didn't see this shown in the results section: *Our scoping review showed that the healthcare sector is increasingly utilising technology to enhance efficiency and improve patient outcomes*	We have deleted this phrase.
	• Not sure what you mean here: *The distributed setting is a complex factor that should be considered in research on distributed teams in the medical context.*	We have also deleted this phrase.

Reviewer 3

Ms. Birgitte Nørgaard, Institute of Public Health, University of Southern Denmark

**Reviewer's comments**		**Authors' response**
**#1**	Abstract: there are a few, yet important, deviations from the PRISMA-ScR guideline recommendations, and I recommend that the abstract is re-structured according to the guideline (e.g., include background information).	Thank you for carefully reading our article and for your useful feedback, which has helped us improve it.<br><br>The abstract now includes a background section, as follows (page 2, lines 26-29):<br><br>**Introduction** The progression of globalisation and technological advancements have led to the emergence of distributed teams in various sectors, including healthcare. However, our understanding of how leadership, communication, and collaboration influence distributed healthcare teams remains limited.

		**Objectives** This study aimed to map knowledge on leadership, communication, and collaboration in short-term distributed teams across various fields to gain insights that could benefit healthcare.
**#2**	As the updated search was conducted more than 1 year before submission, I recommend that the search is updated.	Thank you for suggesting this. We completely agree with your recommendation and have now completed a third updated search, which yielded two additional articles. Interestingly, we observed that many more articles within the healthcare domain were captured in the updated search, even though none of them met our inclusion criteria.
**#3**	Introduction: I really wonder why you include a sentence on teams in industries, business and the military when your review focus on healthcare? I acknowledge that these teams might have been a fact in industries, business and the military prior to healthcare but if this is your message, please clarify.	Thank you for your comment and question. We hope that our response below addresses this concern correctly.<br><br>We understand your concern about including information about distributed teams in various industries and the military in our background information, especially considering that our focus is on the healthcare sector. The purpose of mentioning these other contexts was to highlight the widespread adoption and usage of distributed teams across different sectors and contexts. While our review does indeed focus on the implications for healthcare, we believe it is important to recognise that distributed teams are a broader trend.<br><br>We aimed to contribute to a more comprehensive understanding of the phenomenon in the introduction by including information about the presence of these teams in other sectors. To clarify this, we have revised the sentence to serve as a list of examples of where distributed teams can be found, as follows (page 3, line 71):<br><br>Such teams are becoming increasingly common across various sectors, for instance, in industries, [6] businesses, [7] and the military. [8]
**#4**	Aim: It remains unclear what is meant by "short period" - and why this is of particular interest.	Thank you for this important comment. We have revised the terminology from “short period” to “short-term” to better align with our aim and inclusion criteria. The phrase “short-term” is now used consistently throughout the article, including in the title, which now reads:

		Mapping leadership, communication, and collaboration in short-term distributed teams across various contexts: a scoping review.<br><br>We have also included an explanatory paragraph in the methodology section which elaborates on our choice, as follows (pages 6–7, lines 141–148):<br><br>The short-term criterion was chosen based on our observations during the data search preparation. We noted that many distributed teams in various contexts operate over longer durations (i.e., established teams). This contrasts with healthcare teams, which are often formed for short-term tasks. Short-term teams, which have not collaborated before and plan to disband after finishing the task, work differently than established teams [26] and drawing parallels in teamwork aspects similarly encountered in short-term teams is essential.
**#5**	Methods: My compliments for applying both the framework by Arksey and O'Malley and PRISMA-ScR - I would however, recommend that you apply PRISMA as the overall structure of the methods section - this would in my opinion increase the readability of the section.	Thank you for recognising our application of Arksey and O'Malley's framework and PRISMA-ScR. We appreciate your suggestion to use PRISMA for the overall structure of the methods section to enhance readability.<br><br>At the same time, we wish to retain the headings from Arksey and O'Malley, as they give clarity to our process. However, we have carefully ensured that we have followed the PRISMA recommendations regarding what should be reported in the methods section. Thank you again for your insightful feedback.
**#6**	Methods: You define what is meant by a short period - but it remains unclear and somehow arbitrary that you chose 24 hours. Please elaborate on this and provide references for your choice.	Thank you for raising this concern. We acknowledge that our choice of 24 hours could be viewed as arbitrary. Our aim was to mirror the ad hoc nature inherent in emergency situations within healthcare. In healthcare settings, teams come together to achieve a specific task and disband upon its completion, typically within a constrained timeframe.<br><br>Given that some articles in our review detailed the duration of team interactions, it was important to clarify our definition of "short-term". We do not have a specific reference supporting the exact 24-hour timeframe; rather, this duration reflects the nature of interactions and the tasks commonly encountered within

		shorter timeframes, aligning with how teams operate in healthcare settings.<br><br>Please see our response to your comment #4 for the revised text in the method section.
**#7**	Methods: You state that only one reason for exclusion was used - please mention this reason in the sentence - just for clarity. According to the flowchart, multiple reasons for exclusion were used? this is a bit confusing...	Thank you for noticing this detail and pointing it out. We agree that our previous explanation was confusing, and therefore, we have removed the sentence to avoid further confusion.<br><br>Regardless of the number of reasons for excluding a particular article, only *one* reason for exclusion was listed for each article. This means that when we, the two authors, reviewed an article and identified multiple exclusion criteria (e.g., language and duration), we chose to list only one exclusion criterion for that article (e.g., language). We did not mention other potential criteria that the article may have met.<br><br>However, our flowchart shows exclusion criteria for all excluded articles. Different articles met different exclusion criteria. Each number in the flowchart corresponds to the total number of articles excluded for that specific criterion.
**#8**	Discussion: You mention here barriers and challenges but to my knowledge, this is the first time this is mentioned in the manuscript. Please, do not mention new concepts in the summary of evidence.	Thank you for drawing our attention to the fact that we did not mention barriers at any point before the discussion. Since the discussion has been substantially revised, this sentence has been rephrased as follows (page 17, lines 406-407):<br><br>Over the past twenty years, considerable advances have been made in the literature, shifting focus from early 21st-century difficulties to providing more solutions.
**#9**	Strengths and limitations: You state that you have identified several research gaps - this might be true, but you haven't presented these clearly for the reader - please do so to make this sentence meaningful.	Thank you for pointing this out. We have rephrased the sentence to convey a different interpretation, as follows (page 18, lines 436-437):<br><br>We have presented the evidence reported from various fields and identified a lack of research in healthcare.
**#10**	Flowchart: I find it unnecessarily confusing that you present the results from the two searches	Thank you for this constructive suggestion. Especially now, with three searches, we agree that it would be less confusing to use one figure

	separately and I recommend that this is collapsed.	that consolidates all the searches. We have now revised Figure 1.

Reviewer 4

Prof. Se Young Kim, Changwon National University

**Reviewer's comments**		**Authors' response**
**#1**	I think this is a meaningful study as an examination of teamwork in distributed teams. This appears to be a study that did not directly identify the concept of teamwork, but instead investigated leadership, communication, and cooperation as key variables. If so, further description of the rationale for selecting these variables is necessary in the introduction.	Thank you for your insightful comments and for recognising the significance of our study.<br><br>We appreciate your feedback regarding clarifying our rationale for selecting leadership, communication, and collaboration as key variables. While there are many other potential factors to consider, we selected these three due to their central importance and relevance to teamwork, and because they are common concerns for others studying distributed teams. This choice also lays the foundation for further research, where we will explore these three concepts in empirical studies.<br><br>We have included an explanation of our reasoning in the introduction, as follows (page 4, lines 83-87):<br><br>Understanding the dynamics of leadership, communication, and collaboration is foundational for ensuring effective teamwork in co-located teams, and there is no evidence that distributed teams differ in this respect.[15,20,22] Focusing on these aspects aligns with their established importance in traditional co-located team settings.[15,20]
**#2**	In the results, leadership, communication, and cooperation variables were described separately, but there is some overlap, so there is a need to revise them.	Thank you for this feedback. We have discussed this topic extensively among ourselves, and truly understand your point.<br><br>However, we found it challenging to separate the results, as the aspects are highly interconnected. For example, leadership style often directly influences team communication, and individuals who communicate frequently can emerge as leaders. Our figure also illustrates that many articles simultaneously address multiple interrelated aspects.

		We have revised our results section and hope that the new subheadings will help readers better understand the content.
	The distinction between results and discussion is not clear, and the content of the discussion is relatively lacking, so additional description is needed.	Thank you for this comment, which has helped us to strengthen our article. We agree that the discussion needed a major revision to get the content right. We hope that the revised version meets your expectations.

VERSION 2 - REVIEW

**Reviewer**	**4**
**Name**	**Kim, Se Young**
**Affiliation**	**Changwon National University**
**Date**	**25-Jul-2024**
**COI**	**There are no competing interests.**

I think this is an interesting topic to study. Thank you for your hard work revising the paper. I think the paper has been improved, and I would like to offer some advice.

p.4 However, despite its increased occurrence in healthcare, there is a lack of research on teamwork in distributed healthcare teams.

> There is a need to provide a theoretical basis for these claims.

Distributed leadership is a conceptual and analytical approach to understanding how the work of leadership takes place among the people and in context of a complex organization in Wikipedia. It is necessary to check the definition of the term 'distributed leadership' used by the researcher. There is also a need to check 'distributed communication and collaboration'.

Some contents of 'Applications to healthcare' need to be moved to discussion.

The definition of distributed teams in healthcare is unclear. In order to apply the results to healthcare, please add the description of what characteristics of healthcare institutions are differ from others. Thank you.

VERSION 2 - AUTHOR RESPONSE

Thank you, Editor, for the invitation to submit a major revision. The section below provides a point-by-point response to Reviewer 4.

**Comments by Reviewer: 4**<br><br>**Prof. Se Young Kim, Changwon National University**	**Authors' responses**
I think this is an interesting topic to study.	Thank you, Prof Se Young Kim, for your feedback and for recognising the improvements made to the paper. We

Thank you for your hard work revising the paper. I think the paper has been improved, and I would like to offer some advice.	appreciate your constructive comments and have addressed them as follows:
p.4 However, despite its increased occurrence in healthcare, there is a lack of research on teamwork in distributed healthcare teams.<br><br>> There is a need to provide a theoretical basis for these claims.	We agree and we have added references and a theoretical basis for the claims by referring to Media Richness Theory. Please see page 5, lines 101-104 or the following paragraph:<br><br>Research on human performance and teamwork in distributed teams has been ongoing for years, and various stakeholders and researchers worldwide have studied the topic.[27] However, despite its increased occurrence in healthcare, there is a lack of research on teamwork in distributed healthcare teams.[28] According to Media Richness Theory, which posits that communication effectiveness is enhanced when richer media convey social cues and provide immediate feedback, the complexities of teamwork in distributed healthcare teams may not be fully understood.[29] We therefore conducted a scoping review (ScR) in order to map knowledge on leadership, communication, and collaboration in short-term distributed teams across various fields. This broader perspective could support adaptation in distributed healthcare teams and encourage further research.
Distributed leadership is a conceptual and analytical approach to understanding how the work of leadership takes place among the people and in context of a complex organization in Wikipedia. It is necessary to check the definition of the term 'distributed leadership' used by the researcher. There is also a need to check 'distributed	We agree that the term 'distributed leadership', commonly defined as leadership being understood as an activity dispersed among multiple people within an organization, does not fully capture the geographical aspect that we aim to highlight in our paper. To better align with the focus of our research, which emphasizes aspects of leadership, communication, and collaboration across geographically distributed teams, we believe it is more appropriate to use the subheadings 'Leadership in distributed teams,' 'Communication in distributed teams,' and 'Collaboration in distributed teams.' This terminology better reflects the scope and objectives of our study.

communication and collaboration'.	We have revised the manuscript to replace instances of 'distributed leader' or 'distributed leadership' with 'leader in distributed teams' or 'leadership in distributed teams. ' Similarly, 'distributed communication' has been changed to 'communication in distributed teams' and 'distributed collaboration' to 'collaboration in distributed teams' throughout the document.<br><br>Similar changes have been made in the supplementary material 3: PAGER to ensure consistency with these revisions.
The definition of distributed teams in healthcare is unclear. In order to apply the results to healthcare, please add the description of what characteristics of healthcare institutions are differ from others. Thank you.	Thank you for your valuable suggestion to highlight the unique aspects of healthcare that distinguish it from other sectors. As a minor note, it's essential to acknowledge that the healthcare context is broad, with many variations. However, for the purposes of this study, we have chosen to limit the description as follows: (please see page 4, lines 78-85).<br><br>Unlike other sectors, healthcare faces unique challenges when implementing distributed teams. These challenges include the need for real-time decision-making under time pressure, the critical responsibility of patient care and the formation of ad hoc teams.[14] Healthcare is a dynamic, high-risk environment where team members must quickly adapt, manage complex tasks, and make critical decisions based on rapidly available information. Non-technical skills like leadership and communication are essential for maintaining safety and efficiency.[15] Effective teamwork is crucial for optimising patient outcomes and ensuring safety, as these teams must perform at the highest level.[16,17]
Some contents of 'Applications to healthcare' need to be moved to discussion.	We agree that the section may have a more interpretative or suggestive tone, better suited for the discussion section. We have revised the language to ensure it is more results-oriented and less speculative. These adjustments aim to clearly distinguish between presenting the findings and their

	interpretation. Please see the updated text on page 15, lines 355-361 or as follows:<br><br>**Applications to healthcare**<br><br>The articles underscored the importance of leadership training, given the unique challenges of working in a distributed manner.[40,42,47,62,63,79-81] In healthcare settings, in particular, leaders require training in managing task changes, navigating complexity, fostering collaboration, and effectively utilising technology while supporting team members in its usage.[40,42,82] Additionally, ensuring that isolated team members feel included, involved, and part of the team is crucial for leaders in the distributed team.[69,72] Some studies suggested that richer technology, such as videoconferencing, would be preferable for distributed teams,[26,67,74,75] particularly for complex tasks, as it enables social cues and leads to better intra-team communication and performance.[54,80,83,84] Other research has highlighted the importance of selecting communication technology that aligns with the team's specific needs, as it can impact team effectiveness and communication.[53,80,85,86] Furthermore, one study indicated that adopting an approach following the 'swift trust model,' which assumes that trust is present from the start of the collaboration, can be effective for distributed teams. [61] It has been reported that when trust and a shared mental model is present, distributed teams can achieve outcomes comparable to co-located teams. [43,68]
